# Incidence, risk factors, and outcomes of Fusobacterium species bacteremia

**DOI:** 10.1186/1471-2334-13-264

**Published:** 2013-06-05

**Authors:** Kevin Afra, Kevin Laupland, Jenine Leal, Tracie Lloyd, Daniel Gregson

**Affiliations:** 1 Department of Medicine, University of Calgary, Calgary, Canada; 2 Department of Critical Care, University of Calgary, Calgary, Canada; 3 Department of Pathology and Laboratory Medicine, University of Calgary, Calgary, Canada; 4 Calgary Laboratory Services, Section of Medical Microbiology, #9- 3535 Research Rd. NW, Calgary, Alberta, T2A 2K8, Canada; 5 Infection Prevention & Control, Foothills Medical Centre, Calgary, Canada

**Keywords:** Bacteremia, *Fusobacterium*, Mortality, Incidence, Risk factors

## Abstract

**Background:**

Fusobacterium species (spp.) bacteremia is uncommon and has been associated with a variety of clinical presentations. We conducted a retrospective, population based study to determine the relative proportion of species in this genus causing bacteremia and the risk factors for infection and adverse clinical outcomes.

**Methods:**

All cases of Fusobacterium spp. bacteremia detected at a regional microbiology laboratory serving outpatient and acute care for a population of approximately 1.3 million people over 11 years were identified from a computerized database. Clinical data on these cases was extracted from an administrative database and analyzed to determine underlying risk factors for and outcomes of infection.

**Results:**

There were 72 incident cases of Fusobacterium spp. bacteremia over the study period (0.55 cases/100,000 population per annum). F. nucleatum was the most frequent species (61%), followed by F. necrophorum (25%). F. necrophorum bacteremia occurred in a younger population without underlying comorbidities and was not associated with mortality. F. nucleatum bacteremia was found in an older population and was associated with underlying malignancy or receiving dialysis. Death occurred in approximately 10% of F. nucleatum cases but causality was not established in this study.

**Conclusions:**

Fusobacterium spp. bacteremia in our community is uncommon and occurs in approximately 5.5 cases per million population per annum. F. necrophorum occurred in an otherwise young healthy population and was not associated with any mortality. F. nucleatum was found primarily in older patients with chronic medical conditions and was associated with a mortality of approximately 10%. Bacteremias from other Fusobacterium spp. were rare.

## Background

Fusobacteria are anaerobic gram-negative rods that are a rare cause of serious human disease [1,2]. Two Fusobacterium species, F. nucleatum and F. necrophorum, are the most commonly isolated pathogens within this genus [2,3]. In 1936 Lemierre described cases of oropharyngeal infection followed by septic thrombophlebitis of the internal jugular vein with sepsis syndrome and metastatic disease, typically to the lungs (a syndrome now eponymously named) [4]. F. necrophorum was identified as the causal agent of this condition. Carriage of F. necrophorum in the oropharynx of healthy young adults has been documented, although it may also play a role in non-streptococcal tonsillitis [5]. Serious F. necrophorum infection mostly afflicts young, otherwise healthy, adults [6].

F. nucleatum is a common member of the human oropharyngeal flora, and is an agent in gingival and periodontal disease [7,8]. Of the oral anaerobes, it is also the most likely to cause extra-oral infections [8]. Metastatic infections involving the brain [9,10], liver [11,12], joints [13,14], and heart valves [15] occur albeit rarely. Septic thrombophelebitis in extra-pharyngeal locations has also been documented [16,17]. More recently, Han and colleagues have been exploring the role of F. nucleatum in intrauterine infections and preterm birth [18-20]. Not surprisingly, periodontal disease and dental procedures are frequently identified as the source of invasive F. nucleatum infection. Similarly, chemotherapy induced oropharyngeal mucositis [21,22] and inflammatory bowel disease [23,24] have been implicated in invasive F. nucleatum infection.

Several studies on Fusobacterium infections have previously been published, including hospital [3,25,26] and population based studies [1,2,6]. The largest paper from Hagelskjaer deals specifically with F. necrophorum infections [1]. Huggan et al. [2] primarily looked at non-bacteremic cases and the study by Goldberg et al. is from patients hospitalized at a tertiary care center [3]. As such, the paper by Nohrstrom et al. represents the only population-based study at present that looks at the incidence and diseases associated with Fusobacterium bacteremia as whole [6]. In addition, the relative role of co-morbidities on the acquisition of Fusobacterium bacteremia has not yet been teased out. There is also a wide range of reported mortality: from 5% to 47%. Further clarification of these epidemiological characteristics is best suited to population-based studies. Using such an approach, this study aims to more clearly define the population-based incidence of Fusobacterium bacteremia. We also sought to better elucidate the risk factors, clinical manifestations, and outcomes of Fusobacterium bacteremia.

## Methods

An active, retrospective, population-based surveillance cohort design for the period of January 1, 2000 to December 31, 2011 was used for this study to determine the total incidence, risk factors for, and outcomes of Fusobacterium spp. bacteremia. The cohort studied comprised only residents of the Calgary Zone of Alberta Health Services (AHS), which provides virtually all-medical and surgical care to 1.3 million people in the city of Calgary and a large surrounding area in the Province of Alberta, Canada. Only patients requiring surgery for liver, heart, or lung transplantation are routinely referred elsewhere. More than 95% of all blood cultures collected in both inpatients and outpatients are processed by a single laboratory. Results and basic patient demographic data for all patients were stored in a single laboratory information system. Detailed data (including underlying diagnosis and comorbid conditions) was available from patients requiring hospitalization to any of the 4 major acute care hospitals in a hospital administrative database. This data was linked to create a single surveillance database as previously described by Leal et al. [27]. Data on the use of dialysis is not available for patients less than 18 years of age. Cases not admitted to hospital were used in the calculation of age based rates but not for risk factors or outcome analysis. The Conjoint Health Research Ethics Board at the University of Calgary and Calgary Zone approved this study.

A case of Fusobacterium bacteremia was defined by its isolation from 1 or more sets of aseptically collected blood cultures. No patient had a second episode during the study period, and as such there were no duplicate patients in this study. Isolates confirmed to be Fusobacterium were speciated using standard microbiologic criteria [28] where possible. For those isolates not speciated by standard methods, partial sequencing of the 16S rRNA gene with MicroSeq 500 kits and an ABI Prism 3100 sequencer (Applied Biosystems, Foster City, CA) was performed. Obtained sequences were subject to a BLAST search against the Integrated Database Network System (Smartgene Inc, Raleigh NC) bacterial database to obtain a specific species. Isolates were tested for antimicrobial susceptibility by gradient diffusion methodology (Etest, BioMériuex, St. Laurent QC).

Hospital-acquired (HA) bacteremia was defined as those cases in which the first positive culture was obtained 48 hours or more after hospital admission, or within 48 hours of hospital discharge. Healthcare-associated community onset (HCA) bacteremias were those cases in which the first positive culture was obtained in one of the following circumstances: attended a hospital clinic or emergency department within the previous 5 to 30 days; admitted to a Calgary Zone acute care hospital for 2 or more days within the previous 90 days; resident of a nursing home or long-term care facility; or received outpatient hemodialysis [29]. Community-acquired (CA) bacteremias were those in which the first positive culture was obtained less than 48 hours after hospital admission or were community-onset and not categorized as HCA.

Analysis was performed using STATA/IC 10.0 (StataCorp, College Station TX). Non-normally distributed variables were reported as medians with inter-quartile ranges (IQR). Differences in proportions among categorical data were assessed using Fisher's exact test for pair-wise comparisons. The incidence of Fusobacterium bacteremia was calculated by dividing the number of incident cases by the Calgary Zone population at risk. The population rates for risk factors evaluated in this study were estimated or ascertained from local patient registry data and regional or Canadian survey data as previously described [27,29]. Risks were expressed as incident rate ratios and reported with 95% confidence intervals. For all statistical comparisons, a p-value ≤ 0.05 considered significant.

## Results

During the 11-year study, there were 72 incident cases of Fusobacterium bacteremia. Basic demographic (age, gender, residency) and microbiologic data were available for all cases. Of the ten cases not requiring admission, 3 were F. necrophorum, 5 were F. nucleatum and 2 were unspeciated. Age, gender, and further clinical data was available on 62 patients admitted to one of four acute care hospitals in the region. Among the 72 incident Fusobacterium bacteremias, 35 (49%) were community-acquired, 23 (32%) were healthcare-associated community onset, and 14 (19%) were hospital-acquired. F. nucleatum was identified in 44 cases (61%) and, F. necrophorum in 18 cases (25%). Of the remaining 10 cases (14%), 3 were F. mortiferum, 2 were F. peridonticum, and 5 could only reliably be identified as Fusobacterium spp.

**Figure 1:**
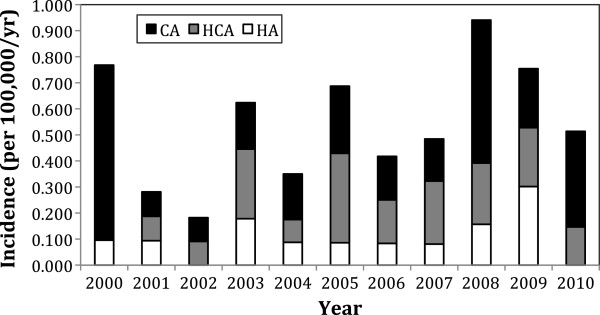
**Annual incidence of Fusobacterium bacteremia by Acquisition Type.** CA = community-acquired; HCA = healthcare-associated community onset; HA = hospital-acquired.

**Figure 2:**
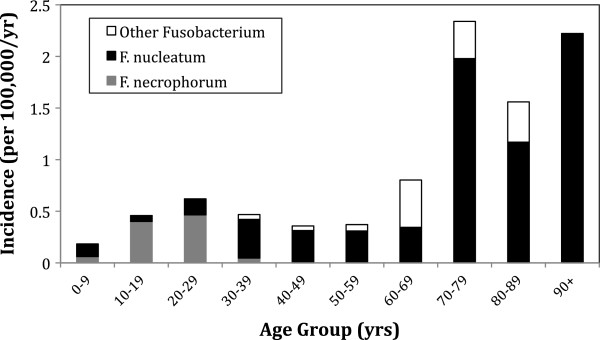
Incidence of Fusobacterium bacteremia by species and age group.

**Figure 3:**
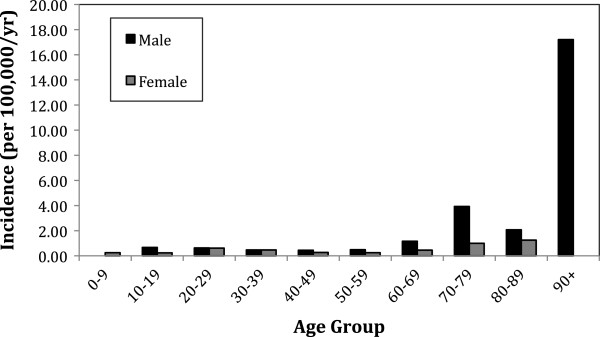
Incidence Rates of Fusobacterium bacteremia by age and gender.

The overall annual incidence of Fusobacterium bacteremia was 0.55 per 100,000 population (Figure 1). Annual variability was seen in keeping with a relatively rare infection, without any clear pattern evident. Incidence of F. nucleatum was 0.34/100,000 and F. necrophorum was 0.14/100,000. Overall median age was 42.1 years. However, a differing pattern of incidence was evident between F. nucleatum and F. necrophorum bacteremia (Figure 2). F. nucleatum cases had a median age of 53.5 years (IQR 36.7-72.0), while F. necrophorum cases had a median age of 21 years (IQR 18.1-23.4). There were no cases of F. necrophorum bacteremia in individuals over the age of 40. Of the 72 incident cases, 45 occurred in men (incidence rate ratio 1.676; 95% confidence interval 1.02-2.81; P = 0.016). Much of the increased risk in men was seen in the elderly (Figure 3).

Among the 62 incident cases with detailed clinical data, 39 were F. nucleatum, 15 were F. necrophorum, and the remaining 8 were other Fusobacterium spp. bacteremia. One or more co-morbidity was present in 39 cases of Fusobacterium bacteremia (63%). The presence of an underlying co-morbidity was more frequently seen in cases of F. nucleatum bacteremia (29/39, 74%) than in cases of F. necrophorum bacteremia (4/15, 27%). Malignancy and dialysis were the greatest risk factors for acquiring Fusobacterium bacteremia (Table 1).

**Table 1:** Risk factors for Fusobacterium bacteremia associated with selected underlying conditions (62 Patients)

**Factor**	Age Group*	**No. (%)**	**IRR (95% CI)**	**p-value**
Malignancy	≥20	10	14.29 (7.28-26.35)	<0.001
Dialysis	≥20	9	14.14 (6.18-28.59)	<0.001
Dementia	≥50	3	4.57 (0.92-14.03)	0.018
Chronic obstructive lung disease	≥12	6	3.46 (1.22-8.02)	0.007
Diabetes	≥1	6	3.24 (1.14-7.51)	0.009
Heart Disease	≥12	7	3.05 (1.17-6.75)	0.007
Alcoholism	≥20	5	2.11 (0.66-5.21)	0.070
Stroke	≥12	1	1.62 (0.04-9.39)	0.296

*IRR* incidence rate ratio, *CI* confidence interval.

*Age Group included in analysis of risk factor.

The primary diagnoses at the time of F. nucleatum and F. necrophorum bacteremia are shown in Table 2. Primary bacteremia, intra-abdominal sepsis, and active hematological disorder were the most common diagnoses in the setting of F. nucleatum bacteremia. F. necrophorum bacteremia was seen most commonly in the setting of primary bacteremia and obstetrical-related infections. Two cases were associated with intravascular thrombus, but it is unclear if this represents Lemierre’s disease as that is not coded within the database.

**Table 2:** Primary diagnosis at time of F. nucleatum and F. necrophorum bacteremia

**F. nucleatum**		**F. necrophorum**	
**Primary Diagnosis (n = 39)**	**Number (%)**	**Primary Diagnosis (n = 15)**	**Number (%)**
No Focus	12 (31)	No Focus	5 (33)
Intra-Abdominal Disease	10 (26)	Obstetrical	3 (20)
Hematologic Disorder	7 (18)	Other ^b^	3 (20)
Other ^a^	3 (8)	Thrombus Associated	2 (13)
Skin and Soft Tissue	2 (5)	Pulmonary	2 (13)
Solid Organ Malignancy	2 (5)		
Genitourinary	2 (5)		

^a^ includes one bone and joint infection, one thrombus-associated infection, and one pulmonary infection. ^b^ includes one skin and soft-tissue infection, one CNS infection, and one intra-abdominal infection.

Susceptibility testing results were available for 70 (97%) isolates. All isolates were susceptible to metronidazole and clindamycin. Penicillin resistance was present in 9% of F. nucleatum isolates and 6% of F. necrophorum isolates.

Overall mortality was 7/62 (11%). There were no deaths in patients with F. necrophorum infection. Four deaths occurred in patients with F. nucleatum bacteremia. The remaining deaths were in patients with other Fusobacterium spp.

## Discussion

The organization of microbiology diagnostic services in the Calgary Zone of AHS provides the ability to study the epidemiology of uncommon infections in this defined population. In the current study, we have found that the overall incidence of Fusobacterium species bacteremia is low at 0.55/100,000 per annum. F. nucleatum bacteremia is over two times more common than F. necrophorum bacteremia (0.34 versus 0.14/100,000 per annum respectively). Population-based incidence rates for Fusobacterium bacteremias are scarce in the literature. Huggan et al. reported their data on invasive Fusobacterium infections from Canterbury region in New Zealand. They found an incidence of 0.99/100,000 per annum [2]. However, their study is not directly comparable as only 17% of their cases had bacteremia. Moreover, very few isolates were identified as F. nucleatum, with most cases being F. necrophorum or remaining unspeciated.

Other studies show data more similar to our findings. A regional study from Finland reported an annual incidence of Fusobacterium bacteremia of 0.55/100,000, identical to our data [6]. In this study the relative frequency of the two common species was equal (0.22 and 0.23/1000 per annum). Similarly, a prospective trial on F. necrophorum bacteremia in Denmark showed an incidence of 0.38/100,000 [1].

Both of these studies found a higher rate of F. necrophorum in their populations compared to our findings. The most likely explanation is year-to-year variability in the number of infections in the population. Another possible explanation for the higher reported incidence in other studies may be due to more consistent species level microbiological diagnosis in these series. Unspeciated isolates comprised 7% of our data set, causing downward bias on our reported species level incidence rates. Though there are some differences on account of methodological and other factors, our data is broadly in alignment with previously reported incidence rates for Fusobacterium bacteremia.

The most striking finding of our study is the absence of F. necrophorum bacteremia in persons over the age of 40, whereas the incidence of F. nucleatum bacteremia increased steadily with age (Table 2). F. nucleatum bacteremia occurred in a group with a median age of 53.5 years compared to 21 years for those with F. necrophorum bacteremia. In our study, other co-morbidities were present in 74% of cases of F. nucleatum bacteremia but in only 27% of those with F. necrophorum bacteremia. Nohrstrom et al. also report results similar to ours: the majority of their cases of F. nucleatum bacteremia were in older patients with co-morbidities. Likewise, F. necrophorum was seen in young patients without co-morbidities [6]. This is consistent with the hypothesis that F. necrophorum bacteremia is primarily a complication of pharyngitis in young adults [5].

Previously published population and hospital-based studies have reported malignancy being a common co-morbidity in those with F. nucleatum bacteremia [3,6,25]. However, our quantification of the role of various co-morbid risk factors on acquisition of Fusobacterium spp. bacteremia is unique in the literature. We report that malignancy and dialysis significantly increase the risk of acquiring Fusobacterium bacteremia. Dementia, chronic obstructive lung disease, diabetes, and heart disease were also risk factors (Table 2).

The underlying diagnosis at the time of bacteremia is shown in Table 2. Intra-abdominal infection (26%) and active hematologic disorders (18%) were the most common primary diagnosis at the time of F. nucleatum bacteremia. A large proportion of F. nucleatum bacteremias did not have a defined focus (31%). A single-hospital study on F. nucleatum bacteremia from Taiwan reported primary bacteremia in 39% of cases and an intra-abdominal source in 12% of cases. However, they had a far higher proportion of respiratory tract sources at 37% [25]. A recent study by Goldberg et al. from the US found a 21% thirty day mortality associated with an increase in the serum creatinine and altered level of consciousness [3]. Persons included in this study were admitted to a tertiary care facility and isolates were not speciated. The nature of the facility in which this study was done and a possible increase in F. nucleatum cases included may account for their higher observed mortality.

No focus of infection was encoded in our database for 33% of F. necrophorum bacteremias. Of those cases that did have a defined focus, 20% were obstetrical, and a combined 26% were thrombotic or pulmonary. These later cases likely represent Lemierre’s syndrome. Unfortunately our administrative data set does not allow us to be definitive. F. necrophorum causing obstetrical infections has been previously reported [2]. We did not see F. nucleatum obstetrical infections as recently described by Han et al. [19]. Somewhat contrasting data from Denmark showed a large group of older patients with F. necrophorum bacteremia from a gastrointestinal or urogenital source [1]. This was not seen in our population.

Our overall mortality was 11% for all Fusobacterium bacteremia, none of whom had F. necrophorum isolated. Other population-based studies also report low mortality rates for Fusobacterium bacteremia: from 1% in New Zealand [2] to 4% in Finland [6]. Yang et al. reported a much higher mortality from a single hospital in Taiwan at 47.4% [25]. This discrepancy is likely due to their study looking at only F. nucleatum bacteremia. In addition, their cases were accrued at a tertiary-care center with nearly half of them requiring ICU admission.

Fortunately, reduced susceptibility to antibiotics was uncommon. None of our isolates showed resistance to metronidazole or clindamycin. Penicillin resistance was present for both F. nucleatum and F. necrophorum, but at low levels. This is in keeping with other studies [25,30].

## Conclusions

Fusobacterium are a rare cause of serious infection. Our data confirms from a population-based perspective that F. necrophorum affects mostly young, healthy adults. In contrast, F. nucleatum affects older individuals with co-morbidities, the highest risk being in persons on dialysis or with underlying malignancy. Fortunately, antibiotic resistance and mortality remains low. Mortality occurs primarily in patients with F. nucleatum, likely due to associated co-morbidities.

## Competing interests

Having any other financial competing interests.

Having any non-financial competing interests (political, personal, religious, ideological, academic, intellectual, commercial or any other) to declare in relation to this manuscript.

## Authors’ contributions

DG and KL developed the regional bacteremia database. DG, KA, and KL were responsible for developing the study purpose and design. TL performed susceptibility testing and identification of the isolates included in this study. JL was responsible for statistical analysis. DG, KA, JL, and KL jointly interpreted data. KA was responsible for the initial draft of the paper. DG, KL, JL, TL and KA all reviewed and substantially contributed to the submitted manuscript. All authors read and approved the final manuscript.

## Pre-publication history

The pre-publication history for this paper can be accessed here:

http://www.biomedcentral.com/1471-2334/13/264/prepub
